# Epithelial-Mesenchymal Transition in Atopy: A Mini-Review

**DOI:** 10.3389/falgy.2020.628381

**Published:** 2020-12-18

**Authors:** Erik D. Anderson, Mohammadali E. Alishahedani, Ian A. Myles

**Affiliations:** Epithelial Therapeutics Unit, National Institute of Allergy and Infectious Disease, National Institutes of Health, Bethesda, MD, United States

**Keywords:** epithelial-mesechymal transition, atopy, atopic dermatitis, asthma, allergic rhinitis, tissue repair

## Abstract

Atopic diseases, particularly atopic dermatitis (AD), asthma, and allergic rhinitis (AR) share a common pathogenesis of inflammation and barrier dysfunction. Epithelial to mesenchymal transition (EMT) is a process where epithelial cells take on a migratory mesenchymal phenotype and is essential for normal tissue repair and signal through multiple inflammatory pathways. However, while links between EMT and both asthma and AR have been demonstrated, as we outline in this mini-review, the literature investigating AD and EMT is far less well-elucidated. Furthermore, current studies on EMT and atopy are mostly animal models or *ex vivo* studies on cell cultures or tissue biopsies. The literature covered in this mini-review on EMT-related barrier dysfunction as a contributor to AD as well as the related (perhaps resultant) atopic diseases indicates a potential for therapeutic targeting and carry treatment implications for topical steroid use and environmental exposure assessments. Further research, particularly *in vivo* studies, may greatly advance the field and translate into benefit for patients and families.

## Introduction

Atopic dermatitis (AD) is the most common inflammatory skin condition in industrialized societies. Symptoms present classically with xeroderma and pruritis. While 85% of cases diagnosed before the age of 5 (1), AD may persist into adulthood (2). The current pediatric prevalence is estimated to be between 11 and 15% in the U.S. (3, 4) and 5–25% globally (5) but, over the recent decades, has been increasing (6). Despite its increasing commonality the etiology of AD is still not fully understood, and standard treatments are not curative. Although most new treatments target the inflammatory component of the disease (7), epidermal barrier dysfunction is an important aspect of AD pathogenesis. Herein, we review the epidermal wound healing mechanisms' (specifically epithelial mesenchymal transition; EMT) established role in allergic rhinitis (AR) and asthma to contrast with the knowledge gaps present in AD. In doing this, we propose that the EMT pathway is a promising therapeutic target for AD and its associated atopic conditions.

## Overview of Epithelial-to-Mesenchymal Transition

Epithelial-to-mesenchymal transition is a process where epithelial cells take on a migratory mesenchymal phenotype (8). Doctor Elizabeth Hay first described the process in the primitive streak of chick embryos (9, 10). Subsequently three main types of EMT have been described which, it should be noted, are defined by the context in which it occurs and not the molecular mediators. Type I occurs during embryo formation and organ development; type II occurs during wound healing, tissue regeneration, fibrosis, and inflammation; type III occurs when neoplastic cells that develop an invasive or metastatic phenotype (11). The initiation event is similar in all the types, with a transition from epithelial associated proteins like E-cadherin, cytokeratin, ZO-1, and Laminin-1 to express mesenchymal associated proteins such as N-cadherin, fibroblast-specific-protein 1 (FSP-1), α-smooth muscle actin, and vimentin (12, 13). The type of EMT relevant to atopic disease is type II (11) given the impact of epithelial turnover and inflammation in the lungs, nasal passages, gut, and skin. *In vitro*, epithelial cells undergoing EMT take on a fibroblastic appearance under light microscopy. The transitioned cells are referred to as fibroblasts or myofibroblasts because of their morphologic and molecular marker changes.

## Chronic Inflammation and Epidermal Barrier Dysfunction in Atopic Dermatitis

While there is no consensus on the etiology of AD, it is generally agreed that it develops through a combination of inflammation and epidermal barrier dysfunction (14). The inflammatory component of disease progression has been stratified into an acute phase (<2 weeks) and a chronic phase (>2 weeks) (15, 16). The acute phase is understood to be primarily driven by T_H_2 cells (17), which release the cytokines interleukin (IL-) IL-4 (18, 19), IL-5, IL-13 (20), and IL-31 (21). During this phase T_H_1 cytokines such as INF-γ, and IL-1β are low, as well as downstream effectors like human β-defensin 2 and 3 and inducible NO synthetase (22–24). The immune profiles of the chronic phase is associated with disparate effects on T_H_2 cytokines such as an increase in IL-5 (25), increased IFN-γ, IL-12 (26), and IL-17A (27), with a decrease in IL-4 (25). An increase in IL-22 secreted by T_H_22 cells progresses through the acute into the chronic phase (24).

Epidermal barrier dysfunction is a major component known to play a role in AD initiation and progression. The epidermis is a stratified squamous epithelium and keratinocytes are the predominant cell type which form the barrier through progressive differentiation. It is histologically divided into four main layers based off of this differentiation pattern, beginning with the stratum basale, moving to the stratum spinosum, granulosum, and ending with the corneum (28). The stratum corneum is made up of terminally differentiated keratinocytes, also known as corneocytes, that have lost their nucleus and form a watertight layer through the formation of an extensive intra and extracellular protein-lipid matrix. Xeroderma is a hallmark of atopic dermatitis, and increased transepidermal water loss has been quantitatively recognized in lesional and non-lesional skin of patients with AD for many decades (29). Given this observation, it is not surprising that the first gene mutation identified to be associated with AD was in the protein filaggrin, an important component of the protein-lipid matrix in the stratum corneum (30). It is a part of the S100-fused type protein (SFTP) family and binds the intermediate filament keratin, which enables its cross linking via transglutaminases and ultimately corneum formation (31). The discovery that some mutations in filaggrin are associated with AD bolstered the theory that barrier dysfunction could be the precipitating event for the condition and cause the inflammatory component through increased antigen sensitization. Additionally, many other proteins and lipids associated with keratinocyte differentiation and epidermal integrity are aberrantly expressed in AD patients (32).

## Epidermal Barrier Dysfunction and the Atopic March

Despite the known role for epidermal dysfunction in AD, EMT has not been directly studied in the disease. However, it has been investigated in other atopic conditions like asthma and allergic rhinitis (AR). Together with food allergy these four conditions make up a progression known as the atopic march. The term stems from the observation that these conditions co-occur with some temporal overlap in many individuals (33). Classically, the atopic march begins with AD in infancy and progresses to food allergy, asthma, and AR. Like AD, these other conditions involve a T_H_2 effector phase at some point in their progression and have barrier dysfunction as a hallmark. Despite the evidence for an epidermal component of AD pathogenesis, most of the new therapies for AD target only the inflammatory component of the disease (7, 14).

## Inflammation Induced Epidermal Barrier Dysfunction

Chronic inflammation may both result from, and contribute to, barrier dysfunction (34). Microvascular permeability is essential for allowing immune cells to traffic to the site of inflammation but comes at the cost of reduced barrier function (35). Similarly, immune cells must traffic to the site of tissue damage in order to perform debridement functions (34). As inflammation persists, uninterrupted signals from inflammatory cytokines may create a positive feedback loop by inducting tissue destruction which releases additional pro-inflammatory danger- and pathogen-associated molecular patterns (36). Therefore, anti-inflammatory treatments may indirectly improve barrier function, even while directly inhibiting EMT. However, while these therapies in combination with hydration are effective, their use can be limited by side effects, lack of long term follow up, limited research in pediatric populations, and high costs (37–41).

## Asthma and EMT

Asthma is one of the most common non-communicable respiratory diseases in the world, affecting ~7.5% of adults (42, 43). While heterogeneous in its pathology, asthma is characterized by intermittent cough, wheezing, and shortness of breath due to lower airway inflammation and/or spasms (42). Increased EMT in bronchial epithelial cells has been hypothesized to contribute to asthma barrier dysfunction (44). Hallmarks of asthma include airway hyperresponsiveness (to triggers such as allergens or exercise) as well as airway remodeling. The remodeling process involves an increase in fibroblasts present in the airway epithelium resulting in a type of fibrosis that may ultimately lead to the obstructive phenotype observed clinically (45). The question of where these fibroblasts originate became an important one for those studying asthma, and EMT served as a possible mechanism to explain this. The growth factor TGF-β, the quintessential stimulant of EMT (46, 47), is increased in the bronchoalveolar fluid of asthmatic patients (48), which suggests EMT could play a role in disease progression (49, 50).

Despite the deluge of subsequent studies on EMT and asthma (Table 1), the topic is not without controversy or limitations. Most of the studies are from *in vitro/ex vivo* cell culture models and therefore may not actually present *in vivo* (115, 116). Additionally, while there are a number of human studies that investigated the relevance of EMT in asthma treatment, vitamin D is the only therapy that has been studied by more than one group (98, 102, 103). However, several animal models have employed lineage tracing to bolster the EMT claim (56, 88). Furthermore, one of the central therapies for asthma, corticosteroids, are potent inhibitors of EMT in additional to being anti-inflammatory (94, 95).

**Table 1:** Summary of publications directly assessing EMT and atopic diseases.

	**Asthma**		**Rhinosinusitis**		**Atopic Dermatitis**	
	***In vitro*/*ex vivo***	**Animal models**	***In vitro*/*ex vivo***	**Animal models**	***In vitro*/*ex vivo***	**Animal models**
Allergen triggers	House dust mites (51, 52) *Dermatophagoides pteronyssinus* (53) Nickel (54)	House dust mites (55) Combustion generated particulate matter (56) Cat dander (57) Mine tailings (58)	*Dermatophagoides pteronyssinus* (53) Fungal sinusitis (59)			
Inflammatory and growth factor triggers	TGF-β (50) SNAIL (60) IL-1β (61) IL-4 and IL-17 (62) IL-22 (63) TSLP (64) Eosinophils (65) Neutrophils (66) TNF-α (67) LIGHT (68, 69) TWEAK (70, 71)	TGF-β (60)	TGF-β (72–75) HIF-1α (76–78) IFN-γ (79)	TGF-β (80)	Periostin (81)	
Other triggers	Compressive stress (82) microRNAs (83)	FIZZ1 (84) microRNAs (85, 86) YKL-40 (87) FGF-10 (88)	WNT3a (89) AGE-RAGE-ERIK pathway (90) Nasal Polyps [associated with EMT markers; (59, 72, 91–93)]			
Treatment targeting EMT	Corticosteroids (94, 95) Propolis (96) Dehydroepiandrosterone (95) Montelukast (97) Vitamin D (98) Diosmetin (99) Procaterol (100)	Kaempferol (101) Vitamin D (98, 102, 103) Ketamine (104) Resveretrol (105) BCG vaccine (106) Azithromycin (107) Aminophylline (108) Anti-natriuretic peptide (109)	Glucocorticoids (94) Arachidonate 15-lipoxygenase inhibition (110) PPAR-gamma agonist (111)	Resveratrol (112)	*Roseomonas mucosa* (113)	*Roseomonas mucosa* (113) Celestrol (114)

*Green text indicates the treatment or marker is associated with induction of EMT, blue text indicates inhibition of EMT. Eosinophilic esophagitis literature contained only assessment of EMT markers on biopsies*.

## Allergic Rhinitis and EMT

Allergic rhinitis is marked by nasal congestion, runny nose, nasal itching, and sneezing (117). AR also has a high incidence, impacting 10–20% of adults in industrialized nations (118). Due to high incidence of co-morbid AR in asthmatics a relatively new model views the two conditions as a one airway disease (119). Despite this overlap between the two conditions, the role that EMT plays in AR is less well studied in asthma, and most of the studies that have been done primarily use chronic rhinosinusitis (CRS) tissue from patients without specifying the etiology (Table 1). This is important because the definition of AR is inflammation of the sinuses specifically due to allergy, whereas chronic rhinosinusitis is inflammation of the paranasal sinuses lasting more than 12 weeks. Although AR can lead to CRS, there are other causes like infection and granulomatous diseases (120). The difference in etiology of CRS could be why the condition can be subtyped into those that develop nasal polyps (CRSwNP) and those that do not (CRSsNP). The inflammatory response associated with nasal polyp development has classically been thought to be driven by T_H_2 polarization verses a T_H_1 response in those without polyps (121). However, some studies have found the picture to be more complex with CRSsNP patients showing T_H_2 polarization (122, 123).

Unfortunately, studies into the role of EMT in AR pathology most often only classify whether EMT markers can be observed in biopsies (59, 72, 91–93). Some studies have found that EMT markers and TGF-β are increased in CRSwNP vs. CRSsNP (72–74, 92), however others have found the opposite (93, 124). Furthermore, like the EMT asthma research, the AR studies are primarily *in vitro* and *ex vivo* models, leaving the need for lineage tracing experiments to determine whether the event is relevant *in vivo*. In addition to TGF-β, Hypoxia Inducible Factor-1α (HIF-1α) may play an important role in AR pathogenesis and is an important inducer of EMT (76–78). Over-expression of the histone acetylase SIRT1, an inhibitor of HIF-1α, may offer possible therapeutic benefit for suppressing NP formation (77). Another therapeutic avenue proposed is resveratrol (105, 112) either in native form or conjugated to a multimeric leucine and lysine rich peptide for enhanced permeability in the nasal epithelium (112).

## Gaps in EMT and Atopic Dermatitis Research

Despite reported links between EMT and the other atopic diseases, few studies have been done on AD and EMT (Table 1). The most direct study that has been done was by Taniguchi et al. who used mouse keratinocytes in an organotypic model to study a role for periostin, a protein whose increased levels in AD correlate with disease severity (81). After finding that periostin could induce EMT, the authors hypothesized a mechanism for how AD epidermal differentiation becomes dysregulated and results in acanthosis. Prior to 2020, only two other publications had looked at the role of EMT in AD: one described triterpene celastrol as a Rac1-medicated inhibitor of EMT (114); the other described EMT markers in lens epithelial tissue from patients with AD associated subcapsular cataracts (125). More broadly, the underlying mechanism that could explain why asthma and AR are associated with enhanced EMT activation whereas AD is associated with reduced EMT activation remains to be elucidated.

## Knowledge Gaps Beyond the Atopic March

Eosinophilic esophagitis (EoE) is an atopic disorder of the esophageal lining believed to be caused by aero- and food-allergen medicated inflammation (126, 127). Although often triggered by similar allergens as the other atopic disorders, EoE is not considered part of the atopic march. EoE is less common than the other atopic disorders, with a prevalence of ~60–120 per 100,000 children (128). Symptoms may range from food aversion and painful swallowing to food impaction and esophageal fibrosis (126). In one study of esophageal biopsies of children, treatment with topical steroids and elemental diets reversed the preexisting significant upregulation of EMT markers including N-cadherin, vimentin, and fibronectin (126). Similarly, biopsy levels of E-cadherin and vimentin were reduced with anti-IL-13 treatment in adults with EoE (127). While these studies add to the link between EMT and atopic disease, direct assessment of EMT in EoE has not been performed.

## Discussion

Atopic dermatitis and its associated diseases asthma and allergic rhinitis represent a substantial burden to the world's health care systems. In this review we consolidated the studies that have tried to identify the role of EMT in AD, asthma, EoE, and AR, to highlight the growing, albeit incomplete, data suggesting a connection between the pathogenesis of these atopic diseases and EMT. They all involve chronic inflammation and epidermal dysfunction, but the underlying cause that sets their progression in motion is still unclear.

A recent publication from our group identified therapeutic benefit of topical microbiome transplantation of *Roseomonas mucosa* from healthy volunteers to the lesions of patients with AD (129). Subsequent research found that lipid mediators from *R. mucosa* stimulate EMT through potentiation of tumor necrosis factor receptor 2 and nicotinic acetylcholine activation; an additional role for flagella interactions with Toll-Like Receptor 5 was identified (113). This finding was consistent with our prior results in autosomal-dominant hyper IgE syndrome, a primary immune deficiency with an eczematous phenotype. In this report we showed dysregulation in EMT (130) downstream of a loss-of-function mutation in *STAT3*. Inhibiting TNF reversed this phenotype, as did treatment with PPAR-γ agonists. Overall, our work is consistent with the literature suggesting that both host and commensal derived lipid-mediators with influence over the ceramide-sphingolipid-arachidonic acid pathway play a role in EMT-related tissue maintenance of the skin (131, 132). However, our work also suggests that the pathologic increase in EMT seen in asthma and AR may be inversed in AD; thus, viable therapies may need to induce EMT in the skin but inhibit EMT in the airways.

The distinction between over-vs. under-active EMT as an underpinning of allergic disease may carry significant consequences. Although originally suggested in 2006, the possibility of “topical steroid addiction” or “topical steroid withdrawal” remains unelucidated (133–135); a PubMed search for (“topical steroid withdrawal” OR “topical steroid addiction”), (“atopic dermatitis” OR eczema) at the time of this manuscript yielded only nine total citations. The main feature of this purported syndrome is that withdrawal of topical corticosteroids leads to greater inflammation than was present prior to steroid application. Although the flare associated with the steroid withdrawal may be treated by resumption of topical steroids, the continued need for treatment may be perceived by the patient as “dependency” and thus “addiction.” While only speculative at this time, the possibility that steroid treatment may alleviate inflammation while worsening underlying defects in EMT is one that warrants further investigation. Such studies must contrast potential for steroid-induced barrier disruption stemming from EMT inhibition against countervailing enhancement of barrier function stemming from reduced inflammation. Furthermore, future work must look into other topical exposures (such as soaps, preservatives, etc.) that may alter EMT functions either directly or indirectly via impacts on commensal organisms. In conclusion, although sparse, the literature on EMT-related barrier dysfunction as a contributor to AD as well as the related (perhaps resultant) atopic diseases indicates a potential for therapeutic targeting. Further research, particularly *in vivo* studies, may greatly advance the field and translate into benefit for patients and families.

## Author Contributions

All the authors meet all criteria for authorship in the ICMJE recommendations, since all authors made substantial contributions to the conception or design of the work, the acquisition and interpretation of data, critical review, and revision of the manuscript and approved the final version. All the authors agreed to be accountable for all aspects of the work.

## Conflict of Interest

The authors declare that the research was conducted in the absence of any commercial or financial relationships that could be construed as a potential conflict of interest.
