# Supplementary figures and images for: Epithelial-Mesenchymal Transition in Atopy: A Mini-Review
Source: Front Allergy. 2020 Dec 18;1:628381. doi: 10.3389/falgy.2020.628381 (PMC8301597; doi:10.3389/falgy.2020.628381)

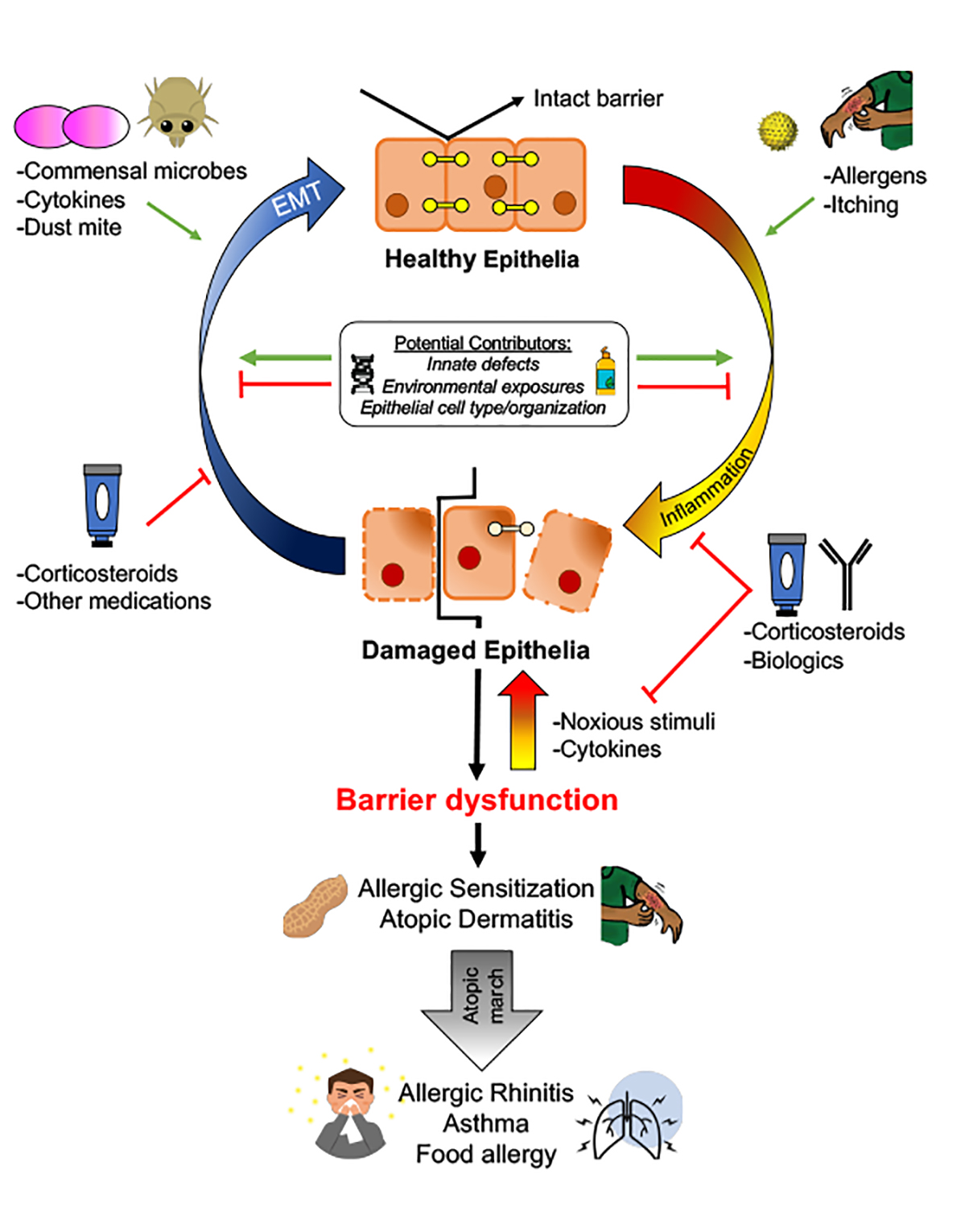

Supplement: Supplementary file 1 [file Image_1.JPEG]
